# Management of Patients With Chronic Pain in Ambulatory Surgery Centers

**DOI:** 10.7759/cureus.10408

**Published:** 2020-09-12

**Authors:** Karina Charipova, Kyle L Gress, Ivan Urits, Omar Viswanath, Alan D Kaye

**Affiliations:** 1 Medicine, Georgetown University School of Medicine, MedStar Georgetown University Hospital, Washington, D.C., USA; 2 Anesthesiology, Beth Israel Deaconess Medical Center, Harvard Medical School, Boston, USA; 3 Anesthesiology, University of Arizona College of Medicine, Phoenix, USA; 4 Anesthesiology, Louisiana State University Health Sciences Center, Shreveport, USA

**Keywords:** enhanced recovery after surgery, pain management, chronic pain, postoperative pain, ambulatory surgery procedures, perioperative care, risk assessment, outpatient

## Abstract

In the setting of increasingly streamlined surgical techniques and perioperative care, the United States healthcare system is seeing a steady rise in the number of procedures being carried out at ambulatory surgery centers. Concurrently, awareness and diagnosis of both chronic pain conditions and substance use disorders have also improved in recent years. As a result of these two shifts, the demographic characteristics of patients undergoing procedures at ambulatory surgery centers are actively evolving. Chronic pain and substance use disorders are difficult to manage in both the outpatient and inpatient settings and present unique challenges in the context of perioperative planning. Both conditions are associated with worsened postoperative outcomes, including refractory pain, decreased functional status, increased length of stay, increased readmission rates, and increased economic costs. There has been a recent movement to include a preoperative risk stratification calculation for these patients, followed by the implementation of enhanced recovery after surgery (ERAS) protocols in these patient cohorts. Taking a step further, patients benefit when standard ERAS protocols are augmented by integrating designated pain specialists into the ambulatory surgery team. This multimodal and multidisciplinary approach must be assessed in the context of the human and financial resources of a given institution and surgery center, but has been shown to improve the quality and safety of perioperative care effectively.

## Introduction and background

Uncontrolled pain is the leading cause of disability in the United States, estimated to affect over 90 million people [1]. Pain incurs physical disability that is frequently accompanied by significant psychological, emotional, and economic burdens [2]. With respect to surgery, uncontrolled pain is not only a risk factor for delayed recovery and various complications but also is a possible adverse event, since surgery is one of the leading causes for the development of chronic pain [3]. It has been estimated that 80% of patients experience moderate to severe pain following surgery [4]. This high prevalence is worrisome, especially given the convention of using opioids for the management of postoperative pain. It is well known that opioid prescription and consumption have been staggeringly increasing in the US over the past two decades. Individuals with chronic pain have been shown to be at a higher risk for opioid misuse and abuse, and there is a significant need for treatment approaches that balance the management of chronic pain and risk for overuse of opioids [5].

The decades in which we have observed a significant increase in opioid use have also seen a steady rise in the prevalence of ambulatory surgery [2, 6]. As the gross number of patients undergoing procedures at ambulatory surgery centers increases, it is likely that patients who suffer from chronic pain will increasingly be part of that population [3]. This likelihood is amplified by the fact that surgical advances and an aging population will simply increase the number of patients with significant comorbidities undergoing procedures [7]. One of the broad issues of surgical pain management is that there are currently no screening instruments or strategies that can reliably and accurately predict patients who are not suitable for opioid therapy or those who require greater monitoring. Presently, the recommendation is to utilize a combination of strategies in the form of opioid assessment screening tools and monitoring programs [5]. Given both the current demographics of patients undergoing ambulatory surgery and the anticipated changes, it is imperative to identify effective protocols by which to accommodate and to ensure the safety of challenging patients.

## Review

The Chronic Pain Patient

Chronic pain is highly prevalent and has historically been managed with daily opioid regimens [8]. It is estimated that as many as 30% of patients presenting for elective surgery are on long-term opioid doses [9]. These patients are at heightened risk for not only opioid tolerance but also hyperalgesia - not ideal given the inherent risks of acute and subacute pain associated with surgery [8]. Chronic opioid use has been consistently associated with difficult-to-manage pain, increased length of postoperative stay, increased readmission rates, and increased economic costs [10]. Studies have shown that in many cases, the severity of preoperative chronic pain can be an accurate predictor of postoperative pain levels [2]. Chronic pain patients undergoing surgery are believed to be at higher risk of developing not only persistent postoperative pain but also postsurgical complications [11]. In these patients, combining chronic opioid use with the effects of general anesthesia and a push for accelerated discharge can lead to serious adverse events such as respiratory depression, delirium, diversion, and overdose [3, 12]. Patients who have a history of chronic opioid use and opioid dependence undergoing treatment for substance use disorders present an added challenge. Ambulatory surgery centers must take care to ensure that these individuals do not develop pain flare-ups or withdrawal symptoms [2].

One of the other issues associated with the treatment of chronic pain patients in ambulatory surgery settings is the potentially overwhelming number of suggestions for pain management. In these patients, recommendations may be made by the patient’s outpatient pain specialist, primary care physician, anesthesiologist, and surgeon. Despite the many benefits offered by multidisciplinary teams, a large number of inputs may predispose to high risk. These recommendations can be especially difficult to manage and optimize during the nearly-universal fifteen-minute preoperative visit conducted by the surgeon or primary care provider [3]. It has been demonstrated that pain management can benefit from standardization, as patients who visited an anesthesiologist for a preoperative consult were found to have decreased postoperative in-house mortality [13]. The use of standardized pain management orders for multimodal intervention for pediatric sickle cell patients has also proven efficacious in decreasing readmission rates [14].

The other well-known issue posed by chronic pain patients when it comes to perioperative care is opioid tolerance. Opioid-tolerant patients have been shown to be at high risk for poor postoperative outcomes. A study comparing opioid-tolerant patients undergoing non-emergent major abdominal surgery to controls found that opioid-tolerant patients were less likely to be compliant with enhanced recovery after surgery (ERAS) protocols and were two times more likely to require readmission. In this study, a patient was deemed “opioid-tolerant” if he or she was taking an opioid regimen equivalent to 60 mg of daily oral morphine for at least one week prior to surgery. The consequences of opioid tolerance include poorly-controlled pain, which in itself, can lead to decreased compliance with ERAS protocols [8]. 

Approach to Care

Pain as a symptom is inherently difficult to manage because it is a subjective phenomenon: the experience of pain is both physiologic and emotional. While the mechanism for sensing pain involves the action of nociceptors coupled with processing in the somatosensory cortex, individual patients have different thresholds for both perceiving and responding to the different components of pain [2]. The notion that the experience of pain depends on many factors beyond the initial insult is sometimes overlooked. For these reasons, patient education is considered one of the key components to the successful implementation of ERAS protocols. Adequate patient counseling is especially important in patients who suffer from chronic pain. For these patients, a plan should be made for both perioperative and post-discharge care well in advance of surgery [15]. During patent counseling, it is important to explain the unfortunate truth that complete relief of pain, especially pain associated with surgery, can sometimes be impossible to achieve. Instead, increasing functional status should be the goal [16]. Managing patient expectations in this manner can enable providers to maximize both outcomes and patient satisfaction.

One of the challenges associated with optimizing pain control in the setting of ambulatory surgery is understanding and anticipating the factors that can complicate postoperative pain. Studies of consecutive ambulatory surgical patients have shown that body mass index, the type of procedure, and the duration of anesthesia are especially helpful in predicting pain outcomes [17]. Orthopedic surgeries have been shown to have a higher incidence of severe postoperative pain when compared to the general, plastic, and urologic surgeries [17]. These results suggest that preoperative risk stratification can facilitate the development of prophylactic and postoperative analgesic regimens. Risk stratification should include a careful pain history that includes the site of pain, duration, frequency, and past medications with dosages. Evaluation of substance use disorder, issues with sleep, and psychiatric history are also recommended [2]. This information can help distinguish several patient risk factors, including tolerance and history of poorly controlled postoperative pain.

The principles of perioperative pain management for patients with chronic pain or substance use disorder and the positive effects of these specialized techniques on morbidity and mortality are well-described in the literature. Unfortunately, it has been demonstrated that these evidence-based protocols are difficult to implement consistently [3]. For this reason, some studies have endorsed the integration of a designated pain specialist as a member of the ambulatory surgery team [2, 3]. Thomas et al. have proposed that the role of this pain physician would be to assess patients who present with a history of chronic pain prior to the day of surgery, develop a specialized pain plan for each patient, and counsel them on realistic expectations. This proposal takes advantage of the reliance of most existing ERAS protocols on a multidisciplinary team. In this setup, the designated pain physician would also be responsible for following up with patients in the days following surgery in order to assess for adverse drug effects and make any necessary changes to the postoperative drug regimen [3]. This early intervention during follow up has the potential to reduce the healthcare burden associated with unnecessary visits to urgent care and emergency rooms [2]. The recommendation is promising given studies that have shown that poor compliance may be one of the factors contributing to less favorable outcomes in chronic pain patients [8]. In fact, it has been demonstrated that appropriate adherence to an ERAS protocol can alone significantly reduce postoperative complications and risk of readmission [3]. Of note, the role of the designated pain physician does not need to be limited to patients with chronic pain or substance use disorder. These individuals can also contribute immensely to the care of patients with implanted intrathecal drug-delivery systems or those who present in acute pain for procedures such as endoscopic retrograde cholangiopancreatography or gastroduodenoscopy, which are being performed at outpatient centers with increasing frequency [2].

Recommendations for Management

One of the major goals of ERAS guidelines is to reduce opioid consumption as much as possible [15, 18, 19]. Studies outlining protocols for ERAS demonstrated the efficacy of multimodal analgesia, regional anesthesia, and structured medication regimens for postoperative nausea and vomiting (PONV) for this purpose. While some multimodal analgesia regimens still implement opioids, it is important to understand their limitations as well as treat their side effects with adjunct medications such as stool softeners and antiemetics [3]. The American Society of Anesthesiologists task force on acute perioperative pain management recommends the use of gabapentinoids, acetaminophen, nonsteroidal anti-inflammatory drugs (NSAIDs), neuraxial, and regional anesthesia whenever possible. Ketamine, which functions as an antagonist of the N-methyl-D-aspartate (NMDA) receptors, has also been shown to decrease postoperative opioid consumption [16]. The goal of multimodal analgesia, especially for chronic pain patients who may use a daily pain regimen, is to take advantage of multiple receptors involved in pain production [3]. Therapy should be catered not only to individual patients but also to the type of surgery-use of gabapentin in combination with a paravertebral block that has been efficacious for breast surgery but cannot be recommended universally [15, 20].

Anxiety associated with surgery can be a significant issue for patients with chronic or uncontrolled pain. For these patients, recommendations have been made for the use of gabapentinoids preoperatively or benzodiazepines perioperatively [21]. Given the complex interplay between psychological state and postoperative outcomes, optimizing control of anxiety can help decrease opioid requirements and risk of poorly controlled pain following surgery [2].

Patients chronically taking opioids as part of their pain regimen should be advised to take their daily dose on the day of surgery to prevent withdrawal. Studies have shown that since patients frequently underreport usage of medications for pain, care must be taken to gather accurate history [2]. Given the effects of tolerance, patients taking chronic opioids may require as much as three times the dosage of perioperative opioids as their opioid-naïve counterparts. Of note, the use of epidural opioids does not circumvent the need to match the systemic effects of chronic oral opioid administration [22]. Thus, if opioids are chosen to be used for surgical pain control, it is imperative to take into account the preoperative daily dose and the opioid requirement for the procedure. If the preoperative daily dose needs to be administered at the surgical facility, long-acting opioids are recommended to achieve steady plasma concentration [2].

Challenges

While widely endorsed in the pain medicine community, the integration of a designated pain specialist into the flow of an ambulatory surgery center is practically challenging. It requires immense planning, assessment of human resources, and financial consideration on the part of the administration. The costs of full-time staffing of at least one fellowship-trained anesthesiologist are significant. Care must also be taken to ensure that this individual is well integrated into the team dynamic and that excellent communication is maintained between perioperative staff members, surgeons, and anesthesiologists. It is important to evaluate the implications of this recommendation critically and to look toward the future to anticipate potential hurdles. Given the increasing numbers of patients undergoing surgery at outpatient facilities, the feasibility that only one pain medicine specialist will be sufficient may be unlikely. 

## Conclusions

There has been a continual increase in the number of procedures being carried out at ambulatory surgery centers. As the sheer number of patients undergoing ambulatory surgery increases, the number of patients who suffer from chronic pain conditions and those who struggle with substance use will follow suit. The management of these patients should include ERAS techniques that emphasize patient education and expectation management as well as the use of multimodal analgesia and regional anesthesia. Further, ambulatory surgery centers should employ pain specialists trained in anesthesia at ambulatory surgery centers to streamline the implementation of ERAS techniques. There is endorsement for the role of this designated physician to serve as a consultant for the surgeons and perioperative staff on site at the ambulatory surgery facility with the aim of increasing safety of both care and discharge.
